# The Association Between the Ct Value of SARS-CoV-2 and the Risk of Death from COVID-19 in Amazonas, Peru, During the Circulation of the Lambda, Gamma, and Delta Variants

**DOI:** 10.3390/v17040558

**Published:** 2025-04-12

**Authors:** Christian J. Campos, Stella M. Chenet, Cecilia Montes-Jave, Fiorella Krapp

**Affiliations:** 1 Dirección Regional de Salud Amazonas, Laboratorio Referencial de Salud Pública Amazonas, Chachapoyas 01001, Peru; christian.campos@upch.pe; 2 Instituto de Investigación en Enfermedades Tropicales (IET), Universidad Nacional Toribio Rodríguez de Mendoza de Amazonas (UNTRM), Chachapoyas 01001, Peru; 3 Facultad de Medicina (FAMED), Universidad Nacional Toribio Rodríguez de Mendoza de Amazonas (UNTRM), Chachapoyas 01001, Peru; 4 Facultad de Medicina de la Universidad Peruana Cayetano Heredia, Instituto de Medicina Tropical Alexander von Humboldt, Lima 150135, Peru; cecilia.montes.j@upch.pe (C.M.-J.); fiorella.krapp@upch.pe (F.K.)

**Keywords:** SARS-CoV-2, COVID-19, viral load, cycle threshold (Ct), death, Peru

## Abstract

This study aimed to assess the association between the cycle threshold (Ct) values of SARS-CoV-2 and the risk of death from COVID-19 in adult patients from the Amazonas region of Peru during the circulation of the Lambda, Gamma, and Delta variants. The study population included both hospitalized and outpatient patients, symptomatic and asymptomatic, between August 2020 and August 2021. The standardized Ct values of the ORF1ab gene were categorized into low and high Ct groups based on the median Ct value (28.4). Mortality data within 60 days were obtained from the Peruvian epidemiological surveillance system (Notiweb). The risk of death was estimated using Cox regression, adjusting for relevant predictors and potential confounding variables. Among symptomatic COVID-19 patients, no significant difference in the risk of death was observed between those with low and high Ct values (adjusted hazard ratio [aHR] = 1.61; 95% confidence interval [CI], 0.97–2.67; *p* = 0.067). However, hospitalized patients with low Ct values had a significantly higher risk of death compared to those with high Ct values (aHR = 1.82; 95% CI, 1.06–3.12; *p* = 0.030). This association persisted after adjusting for age, sex, occupational group, symptom duration, comorbidities, and epidemic dynamics. In conclusion, while Ct values in symptomatic COVID-19 patients (both hospitalized and outpatient) are not associated with a 60-day mortality risk, a low Ct value is linked to an increased risk of death in hospitalized patients.

## 1. Introduction

The SARS-CoV-2 virus (severe acute respiratory syndrome coronavirus 2) has resulted in over 6 million deaths worldwide, including more than 219,448 deaths in Peru [1]. Amazonas, a region in northeastern Peru, consists of seven provinces with an approximate population of 426,806 inhabitants [2]. As of March 2023, the region reported 1409 deaths, yielding a fatality rate of 2.75% [3]. The first case of COVID-19 in Amazonas was reported on April 8, 2020, with the first wave peaking at the end of July 2020, recording over 3400 cases per week [4]. The second wave, which lasted from late 2020 to the end of 2021, was dominated by the Lambda variant [5].

The confirmatory diagnosis of COVID-19 is typically made via the quantitative reverse transcription polymerase chain reaction (qRT-PCR), a nucleic acid amplification test considered the gold standard for detecting specific viral sequences in the E, ORF1ab, N, and S genes. The nasopharyngeal or oropharyngeal swab, collected in a tube with the viral transport medium (VTM), is the most commonly used sample for diagnosis [6]. The ORF1ab gene is known to detect SARS-CoV-2 with high sensitivity [7].

qRT-PCR results report the cycle threshold (Ct) value, which represents the cycle number at which the detection of viral genetic material becomes positive. The Ct value is inversely related to the amount of viral RNA in the sample, meaning that a lower Ct value corresponds to a higher viral load [8,9]. Viral load refers to the number of viral copies per milliliter (ml) of the sample. Each increase of approximately 3.3 Ct represents a tenfold reduction in viral RNA [8,9], and both the Ct value and viral load provide insights into the amount of viral RNA present in the sample [9].

Currently, no diagnostic kits are available with standardized reference materials to directly quantify the viral load in copies/mL for SARS-CoV-2, nor are there established Ct value cutoffs for categorizing viral loads as high or low [10]. Previous studies have estimated the SARS-CoV-2 viral load by constructing standard curves to extrapolate Ct values to copies/mL [11,12], while others have grouped Ct values into categories such as high, medium, and low based on their findings [13,14,15].

Several risk factors have been identified as being associated with mortality from COVID-19, including age [16,17], cardiovascular disease [18], obesity [19], diabetes [19], chronic respiratory diseases [20], and pregnancy [21]. The SARS-CoV-2 viral load has also been suggested as a potential risk factor for the severity and mortality of COVID-19. A high viral load may be linked to an exaggerated immune response, leading to the hyperinflammatory state observed in severe COVID-19 cases [22]. Furthermore, patients with compromised immune systems may experience higher viral loads, which could contribute to a more severe progression of the disease [14,23]. Studies in the United States and Japan have found higher viral loads in symptomatic patients compared to asymptomatic ones [24,25]. An observational study involving hospitalized patients demonstrated an independent association between viral load and mortality, with a 7% increase in the risk of death for each log-transformed increase in viral load [11]. Other studies from the United States and the United Kingdom also found a significant association between viral load (as estimated from the Ct value) and mortality, with odds ratios (ORs) as high as 6.05 for patients with a high viral load [13,14,26]. However, studies from Chile and Italy observed similar viral loads in severely ill and mild patients [12,27], suggesting that viral load may be more closely related to the duration of illness [26].

Discrepancies between studies may arise from differences in study populations or variations in viral kinetics associated with different SARS-CoV-2 variants [28].

The objective of this study was to assess the association between the Ct value (an indirect estimate of viral load) of SARS-CoV-2 and the risk of death from COVID-19 in adult patients from the Amazonas region of Peru during the circulation of the Lambda, Gamma, and Delta variants.

## 2. Materials and Methods

### 2.1. Study Design, Population, and Sample

**Figure 1:**
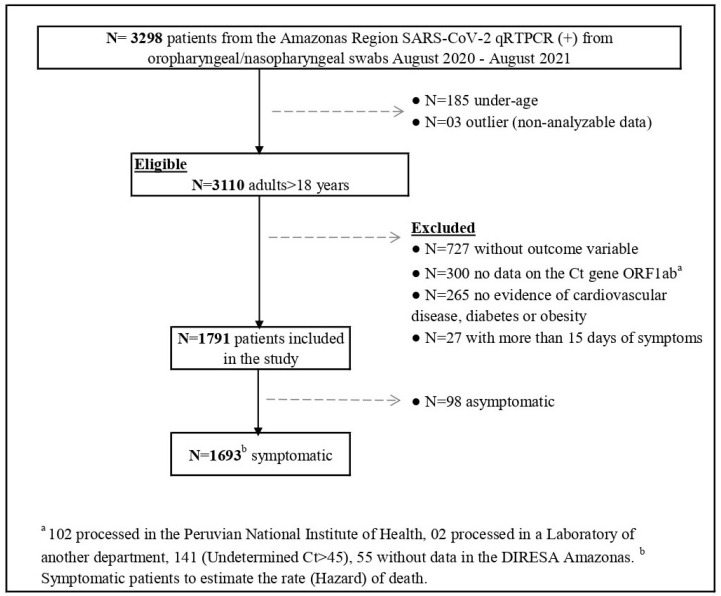
Flow diagram of patients diagnosed with SARS-CoV-2 through qRT-PCR between August 2020 and August 2021 included in the study and reasons for exclusion. Amazonas, Peru.

The study population included individuals over 18 years of age, both hospitalized and outpatient, symptomatic and asymptomatic, who were registered in the epidemiological surveillance system database (Notiweb) with a 60-day follow-up period and a positive molecular diagnosis of SARS-CoV-2 from the Laboratorio de Referencia Regional de Salud Pública (LRRSP) of the Dirección Regional de Salud Amazonas (DIRESA) during the period from August 2020 to August 2021. The LRRSP is responsible for diagnosing communicable and non-communicable diseases of public health importance. If a patient had multiple positive results, only the first sample was included in the analysis. Patients were excluded if they lacked outcome data, had no registered Ct value for the ORF1ab gene, were missing information on significant comorbidities, or had symptoms for more than 15 days at the time of molecular testing. Additionally, patients with outlier data for the Ct value were excluded. (Figure 1)

SARS-CoV-2 diagnosis was performed using nasopharyngeal or oropharyngeal samples collected in universal transport medium (UTM) (COPAN Diagnostics Inc., Murrieta, CA, USA). RNA was extracted using silica columns. Detection was carried out via qRT-PCR with kits approved by the Dirección General de Medicamentos, Insumos y Drogas (DIGEMID) using a QuantStudio 5 thermal cycler.

**Figure 2:**
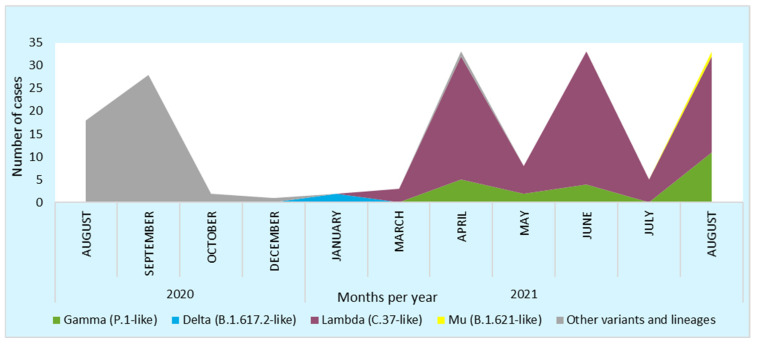
Distribution of SARS-CoV-2 variants in the Amazonas region during August 2020 to August 2021.

The study period coincided with a decline in cases following the first wave, during which no variants of concern or interest were circulating. It also encompassed the entire second wave of infections in the Amazonas region, during which the Lambda variant predominated, with the Gamma variant circulating to a lesser extent [5] (Figure 2). COVID-19 vaccination started with vaccination to healthcare workers in February 2021, followed by the general population in July 2021 [4,5]. This period was marked by the highest case fatality rate observed in the region [4].

### 2.2. Data Management and Statistical Analysis

The cycle threshold (Ct) value of the ORF1ab gene was obtained from the LRRSP registry of samples processed for COVID-19 diagnosis. However, due to the use of five different kits (RADI-COVID-19, Zybio, Allplex^TM^, Biotechrabbit, and Norgen Biotek), each with varying ranges and means, the Ct values were standardized by the mean Ct for each kit. Standard deviations (SDs) were used to improve the distribution of this variable. A linear regression analysis was conducted to correlate the data with the Ct values, and the corrected Ct values, referred to as Cts (standardized Ct), were derived using the linear regression equation. These standardized Ct values exhibited a distribution similar to that obtained through mean standardization.

The epidemic dynamics of the cases were stratified based on the parameters used by the US Centers for Disease Control and Prevention (CDC) to calculate community transmission levels, as well as the wave periods defined by the Oficina de Epidemiología, Prevención y Control de Enfermedades of the Amazonas DIRESA. Data on death at 60 days were extracted from the Notiweb database. Data collected included symptom duration at the time of molecular testing, sample collection date, age, sex, race or ethnicity, hospitalization status, and self-reported comorbidities, such as pregnancy, cardiovascular disease, diabetes, obesity, HIV, chronic kidney disease, chronic lung disease, and cancer. After selecting the relevant variables, they were compiled into a new database and imported into the Stata statistical software package.

Univariate analysis was performed to descriptively characterize the data according to the type of variable, reporting percentages and means with their respective dispersion measures. A bivariate analysis was conducted to identify variables associated with an increased risk of death using the chi-square test (χ^2^) for categorical variables and the Mann–Whitney U test for continuous variables. The Ct value in relation to the duration of illness was assessed by examining the Ct values according to the number of symptom days and presented in a scatter plot with a smoothed curve.

To estimate the hazard rate for death from COVID-19 based on the Cts value, proportional hazards analysis was performed using multivariate Cox regression, with the Breslow method for ties. This analysis was conducted separately for symptomatic COVID-19 patients and hospitalized patients. Due to the non-linear nature of the Ct values, which varied according to symptom duration, the Cts values were first stratified. The median Ct value of 28.4 was chosen as the cutoff point, categorizing Cts values into low (<28.4) and high (≥28.4) groups. This cutoff was similar to the values estimated by Magleby et al. [13] and Tanner et al. [15]. Confounding variables were included in the model, and the proportional hazards assumption was verified using appropriate tests.

Additionally, Cts values were compared between symptomatic and asymptomatic patients using multivariate logistic regression, adjusting for confounding variables. For logistic regression, Cts values were stratified in the same manner as in the Cox regression analysis. The model’s assumptions were evaluated for unusual values using Pearson’s residuals, Pregibon’s dbeta, and deviance residuals. All analyses were conducted using Stata version 17.0 statistical software, and a *p*-value < 0.05 was considered statistically significant.

This research was registered on the Proyectos de Investigación en Salud (PRISA) platform (code EI00000003229) and was approved by the Comité Institucional de Ética en Investigación (CIEI) of the Universidad Peruano Cayetano Heredia (UPCH) under registration code 207259.

## 3. Results

A total of 1791 patients who tested positive for SARS-CoV-2 by qRT-PCR and met the inclusion criteria during the study period (08/2020–08/2021) were identified (Figure 1). The median age of the cohort was 40 years [IQR: 29–54], and 903 (50.4%) were male.

The survival and dynamics of the Cts values over the course of illness were evaluated exclusively in symptomatic patients (*n* = 1693). In the population with symptomatic COVID-19, there were 67 (3.9%) fatal outcomes, and in hospitalized patients, there were 65 (23.8%). The median time to death in both groups was 15 days [IQR: 12–22].

Cts values varied in relation to epidemic dynamics, with the lowest values observed during periods of high transmission in the first wave (27.9 [IQR: 22.7–33.8]) and the highest values during the decline of the first wave when transmission was lower (28.9 [IQR: 23.9–35.3]). At the onset of the second wave, during a phase of low transmission, the Cts values were 28.4 [IQR: 23.3–34.8], whereas during the peak of the second wave, when transmission was high, the values decreased to 27.4 [IQR: 22.3–33.7]. During the decline of the second wave, as transmission decreased, the Cts values increased to 30.3 [IQR: 25.5–33.7].

Bivariate analysis identified several factors most strongly associated with fatal outcomes in symptomatic COVID-19 patients, including age, sex, cardiovascular disease, diabetes, and epidemic dynamics. In hospitalized patients, age showed a stronger association with fatal outcomes (Table 1).

**Figure 3:**
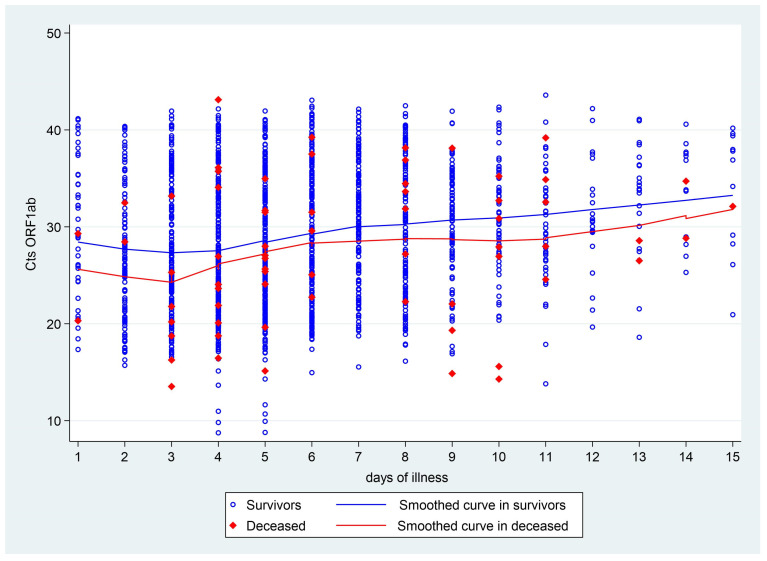
Cts ORF1ab values of SARS-CoV-2 depending on the time of illness in the Amazonas region. The trend line of the Cts ORF1ab value over the time of illness was stratified according to the outcome, in blue (survivors) and in red (deceased). Blue circles indicate the individual ORF1ab Cts of the surviving patients. The red diamonds indicate the individual Cts of the deceased patients.

The smoothed curve of the Cts of SARS-CoV-2 revealed a trend of decreasing Cts values until the third day of symptoms, followed by an increase until day 7, and then a gradual rise thereafter. These changes throughout the course of the infection were observed in both deceased and surviving patients (Figure 3).

**Figure 4:**
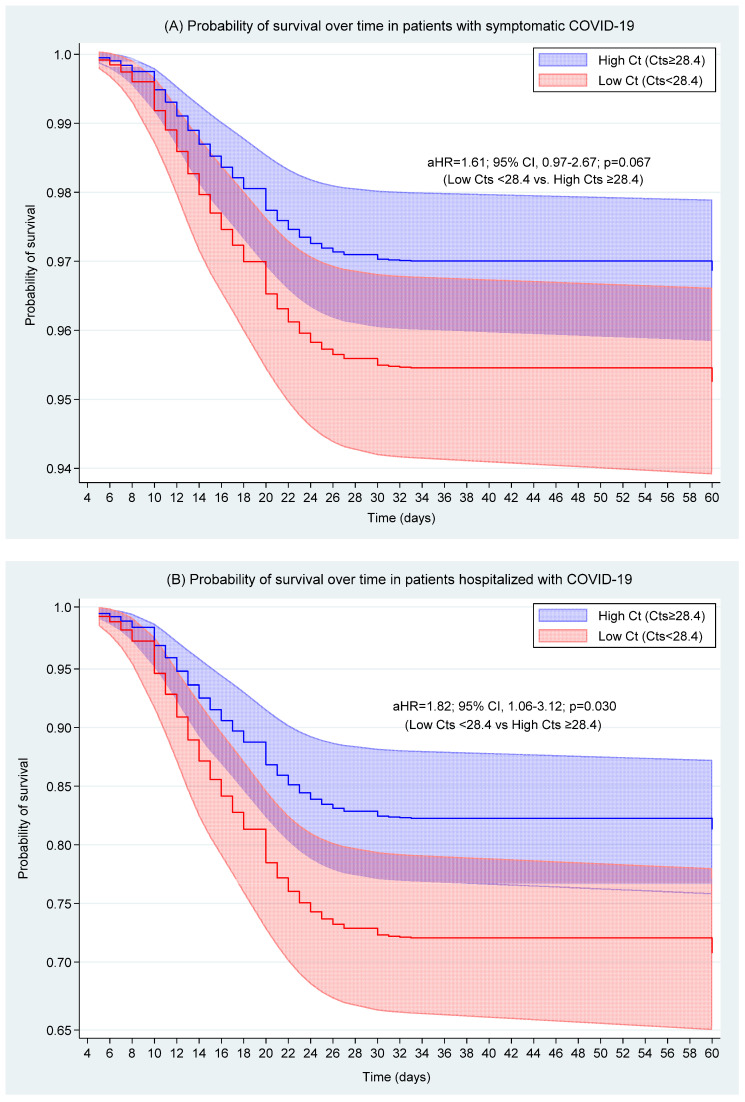
Probability of survival over time in patients from the Amazonas region with COVID-19 stratified by Ct value. Ct values were categorized into high Ct (Cts ≥ 28.4) and low Ct (Cts < 28.4). (**A**) Patients with symptomatic COVID-19 and (**B**) hospitalized patients with COVID-19.

In patients with symptomatic COVID-19, the Hazard Ratios (HRs) of death adjusted for age, sex, occupational group, days of symptoms, comorbidities, and epidemic dynamics were higher in patients with low Ct (Cts < 28.4) compared to those who had high Ct (Cts ≥ 28.4), but this association was not statistically significant (aHR = 1.61; 95% CI, 0.97–2.67; *p* = 0.067) (Figure 4A and Table 2).

In hospitalized patients, the Hazard Ratios (HRs) of death adjusted for age, sex, occupational group, days of symptoms, comorbidities, and epidemic dynamics were higher in patients with low Ct (Cts < 28.4) compared to those with high Ct (Cts ≥ 28.4) (aHR = 1.82; 95% CI, 1.06–3.12; *p* = 0.030) (Figure 4B and Table 2).

In our logistic regression model, we found that, compared to those with high Ct, having a low Ct was independently associated with being a symptomatic patient (aOR = 2.57; 95% CI, 1.61–4.07; *p* < 0.001).

## 4. Discussion

This study evaluated the association between the Ct value of SARS-CoV-2 and the risk of death from COVID-19 in a northeastern region of Peru during the period characterized by the highest fatality rates, which coincided with the introduction of the Lambda variant and its co-circulation with the Delta and Gamma variants [5]. Over 1700 patients, both hospitalized and outpatients, diagnosed with COVID-19 through molecular testing were included in the analysis.

Our findings indicate that the Ct value of SARS-CoV-2 (which is inversely proportional to viral load) in nasopharyngeal/oropharyngeal samples changes throughout the course of the infection, with lower values (higher viral load) observed during the early days of symptoms. This observation is consistent with other studies, indicating that Ct values are lower at the onset of symptoms [29,30], or shortly thereafter [24,30]. Wölfel et al. [31] even report that the maximum viral load (low Ct) was reached before the fifth day of infection, while in our study, the lowest Ct values were found around the third day. This slight discrepancy could be attributed to differences in the study populations, as Wölfel et al. [31] studied a European, younger cohort without underlying conditions, in contrast to the population in our study. After reaching the lowest Ct value (maximum viral load), Ct values progressively increase [30], a trend also observed in our study. These dynamics remained consistent throughout the disease course, regardless of patient outcomes, demographic characteristics, comorbidities, or the context of the pandemic. As previous studies were conducted in various countries with different demographics and likely circulating variants, it is clear that the use of Ct as an indirect measure of viral load must take into account the dynamic changes in the course of infection.

This study assessed the hazard rate of death based on the Ct value of SARS-CoV-2 using a proportional hazards model in a large cohort of symptomatic patients, including both hospitalized and outpatient individuals. No significant association was found between the risk of death and the Ct value in the broader population. Our results differ from studies conducted in Japan [25] and the USA [22,26]. However, it is important to note that in two of these studies [22,25], the association found was based on a group comparison without adjusting for COVID-19 mortality predictors. In the third study [26], a high risk of mortality was observed in patients with Ct < 22 (aOR = 4.2; 95% CI, 1.62–10.86; *p* = 0.01), but the analysis was only adjusted for HIV status, age, and sex. Furthermore, the population consisted of 245 patients, 237 of whom were hospitalized, limiting the generalizability of these findings to hospitalized patients alone.

In the subgroup of hospitalized patients, we found a significant association between low Ct values (Ct < 28.4) and increased mortality risk (aHR = 1.82; 95% CI, 1.06–3.12; *p* = 0.030), indicating an 82% increase in the risk of death. These findings are consistent with previous studies showing that higher viral loads, estimated from low Ct values or measured in copies/mL, are associated with a higher risk of mortality in hospitalized patients [11,13,14,15]. Tanner et al. [15] demonstrated that low Ct values (high viral load) are linked to higher mortality, while Westblade et al. [14] found that patients with a high viral load (Ct < 25) had a hazard ratio (HR) of 5.20 (95% CI, 1.65–16.44; *p* = 0.005) compared to those with a low viral load (Ct > 32). Similarly, Magleby et al. [13] reported that a high viral load (Ct < 25) was associated with an HR of 5.06 (95% CI, 2.86–8.96; *p* < 0.001) compared to those with a low viral load (Ct > 30). The high HR and wide CI observed in these studies may be due to the higher prevalence of comorbidities (three times higher than in our study) and the presence of older patients, as high viral loads are associated with older age and comorbidities, both of which increase the risk of mortality. Additionally, these studies did not adjust for mortality predictors or the duration of the illness, which could affect the results.

The upper limit of the confidence interval (CI) for the adjusted hazard ratio (aHR) of 3.12 is of clinical significance, suggesting a more than threefold increased risk of death for patients with a Ct value ≤ 28.4. However, this analysis was conducted within a subgroup of our population, where an association was observed despite limited statistical power (power = 45.1). These findings underscore the need for further investigation in hospitalized populations, particularly through larger, multicenter studies, to more accurately determine the magnitude of this effect.

Vaccines have played a critical role in reducing COVID-19 mortality, and some studies suggest that vaccination also decreases viral load [32,33]. As the general population had been largely vaccinated by the conclusion of this study, vaccination did not influence our results, unlike in the studies discussed previously. To assess the potential impact of vaccination, we compared our findings with later studies conducted after widespread vaccine administration.

Two studies from Mexico, involving large cohorts of both hospitalized and outpatient individuals, provide relevant comparisons. In the first study, 18.6% of participants had a history of vaccination, and the researchers documented that mortality was higher in patients with a high viral load (Ct < 25) [34]. In the second study, 25.6% of individuals were fully vaccinated. In this cohort, disease severity was not correlated with a higher viral load, and no significant differences were found between the viral loads of vaccinated and unvaccinated patients. The authors also suggested that co-infections and other immunological factors [35], such as antibody dynamics [36], lower immunogenicity [37], and cellular responses [38], may influence patient outcomes.

In Israel, a cohort study of 152 hospitalized patients, all of whom had high comorbidity rates and were fully vaccinated with the Pfizer/BioNTech BNT162b2 vaccine, found that a very high viral load was associated with an increased risk of poor outcomes [39]. Similarly, in India, viral load in hospitalized patients was consistently found to be associated with an increased risk of mortality. Although vaccinated patients generally exhibited lower viral loads, high viral loads remained associated with increased severity of infections caused by the Delta (B.1.617.2) and Omicron (BA.1) variants of SARS-CoV-2 [40].

Overall, the results before and after the widespread vaccination campaign are consistent. There is no clear consensus on the relationship between viral load and clinical outcomes in the general population, which includes both outpatients and hospitalized patients. However, the studies referenced herein show a greater consensus that a high viral load is associated with unfavorable outcomes in hospitalized patients.

Additionally, we observed that symptomatic patients had lower Ct values compared to asymptomatic patients. This finding aligns with previous studies by Salvatore et al. [24] and Tsukagoshi et al. [25]. In contrast, Mattar et al. [41] did not find a significant difference in viral load between symptomatic and asymptomatic patients, although viral loads tended to be higher in symptomatic individuals. This lack of significance could be due to the small sample size in Mattar et al.’s study (*n* = 35).

Several limitations of the study should be noted, including the reliance on secondary databases that lacked patient-specific information on SARS-CoV-2 variants and vaccination status, the latter due to the lack of widespread vaccine availability during the period analyzed. There is also a possible underreporting of positive cases and variations in access to medical care. Differences in the timing of patient care and access to COVID-19 diagnosis via qRT-PCR, as well as limitations in materials and human resources, also constrained the study. The study period and local disease dynamics were considered during analysis to mitigate these limitations. While Ct values from qRT-PCR are an indirect measure of viral load, alternative methods such as the standard curve and digital PCR offer more direct quantification of viral RNA copies. However, qRT-PCR remains a widely accessible and routinely used tool, enhancing the practical applicability of our findings.

In conclusion, our study provides valuable insights into the dynamics of SARS-CoV-2 Ct values over the course of infection and their potential clinical relevance, particularly in hospitalized patients. The Ct value at the time of hospital admission could serve as an additional clinical marker to aid in the prediction of COVID-19 mortality, complementing established predictors. However, to maximize the clinical utility of the Ct value, further efforts are needed to standardize diagnostic platforms, improve interlaboratory reproducibility, and develop reference data specific to our population. Larger, multicenter studies are required to establish universal Ct cut-off points with greater precision.

## 5. Conclusions

In the present study, we conclude that the Ct value of SARS-CoV-2 (an indirect measure of viral load) in adults with symptomatic COVID-19, including both hospitalized and outpatient cases, is not associated with survival. However, in hospitalized patients, the Ct value is significantly associated with the risk of death, even after adjusting for age, sex, occupational group, symptom duration, comorbidities, and epidemic dynamics.

## Figures and Tables

**Table 1:** Characteristics of COVID-19 patients diagnosed by qRT-PCR according to clinical outcomes. Amazonas, Peru, during the period of 2020–2021.

	Patients with Symptomatic COVID-19 (Outpatients and Hospitalized)				Hospitalized Patients				Outpatients			
	Total (*n* = 1693)	Survivors (*n* = 1626)	Deceased (*n* = 67)	*p* Value ^a^	Total (*n* = 273)	Survivors (*n* = 208)	Deceased (*n* = 65)	*p* Value ^a^	Total (*n* = 1420)	Survivors (*n* = 1418)	Deceased (*n* = 2)	*p* Value ^a^
**Demography**												
Age in years ^b^	40 [29–54]	39 [29–53]	67 [58–82]	<0.001	55 [44–68]	52 [41–64]	66 [58–81]	**<0.001**	37 [28–50]	37 [28–50]	88 [87–89]	<0.015
Ethnicity												
Mestizo	1692 (99.9%)	1625 (99.9%)	67 (100%)	0.839	273 (100%)	208 (100%)	65 (100%)	NA	1419 (99.9%)	1417 (99,9%)	2 (100%)	0.97
Indigenous	1 (0.1%)	1 (0.1%)	0 (0%)		0 (0%)	0 (0%)	0 (0%)		1 (0.1%)	1 (0.1%)	0 (0%)	
Sex												
Male	849 (50.2%)	800 (49.2%)	49 (73.1%)	<0.001	176 (64.5%)	129 (62%)	47 (72.3%)	0.13	673 (47.4%)	671 (47.3%)	2 (100%)	0.1136
Female	844 (49.8%)	826 (50.8%)	18 (26.9%)		97 (35.5%)	79 (38%)	18 (27.7%)		747 (52.6%)	747 (52.7%)	0 (0%)	
Pregnancy	12 (0.7%)	12 (0.7%)	0 (0%)	0.48	2 (0.7%)	2 (1%)	0 (0%)	0.427	10 (0.7%)	10 (0.7%)	0 (0%)	0.905
Occupational group												
High level of exposure to SARS-CoV-2 ^c^	489 (28.9%)	473 (29.1%)	16 (23.9%)	0.357	64 (234 %)	48 (23.1%)	16 (24.6%)	0.798	425 (29.9 %)	425 (30%)	0 (0%)	0.355
Low level of exposure to SARS-CoV-2	1204 (71.1%)	1153 (70.9%)	51 (73.1%)		209 (76.6%)	160 (76.9%)	49 (75.4%)		995 (70.1%)	993 (70%)	2 (100%)	
**Comorbidities**												
Cardiovascular disease	132 (7.8%)	119 (7.3%)	13 (19.4%)	<0.001	50 (18.3%)	38 (18.3%)	12 (18.5%)	0.972	82 (5.8%)	81 (5.7%)	1 (50%)	0.007
Diabetes	54 (3.2%)	47 (2.9%)	7 (10.5%)	0.001	17 (6.2%)	10 (4.8%)	7 (10.8%)	0.083	37 (2.6%)	37 (2.6%)	0 (0%)	0.817
Obesity	25 (1.5%)	25 (1.5%)	0 (0%)	0.307	11 (4%)	11 (5.3%)	0 (0%)	0.058	14 (1%)	14 (1%)	0 (0%)	0.888
Other comorbidities	18 (1.1%) ^d^	15 (1%)	3 (4.5%)	NA	8 (3%) ^d^	6 (2.9%)	2 (3%)	NA	10 (0.7%) ^d^	9 (0.6%)	1 (50%)	NA
**Laboratory data**												
Ct_s_ value gene ORF1ab ^b^	28.4 [23.4–34.5]	28.5 [23.5–34.6]	27.9 [22.0–33.2]	0.17	28.9 [23.4–33.6]	29.5 [23.7–33.7]	27.2 [22.0–32.7]	0.185	29 [24.1–34.4]	29 [24.1–34.5]	31.2 [29.8–32.7]	0.602
High Ct (Ct_s_ ≥ 28.4)	847 (50%)	816 (50.2%)	31 (46.3%)	0.53	146 (53.5%)	117 (56.3%)	29 (44.6%)	0.101	701 (49.4%)	699 (49.3%)	2 (100%)	0.152
Low Ct (Ct_s_ < 28.4)	846 (50%)	810 (48.8%)	36 (53.7%)		127 (46.5%)	91 (43.7%)	36 (55.4%)		719 (50.6%)	719 (50.7%)	0 (0%)	
**Epidemic dynamics**												
First wave_High transmission	155 (9.2%)	150 (9.2%)	5 (7.5%)	<0.001	27 (9.9%)	22 (10.6%)	5 (7.7%)	0.117	128 (9%)	128 (9%)	0 (0%)	0.939
Decline of the first wave_Low transmission	88 (5.2%)	82 (5.0%)	6 (8.9%)		22 (8.1%)	16 (7.7%)	6 (9.2%)		66 (4.7%)	66 (4.7%)	0 (0%)	
Beginning of the second wave_Low transmission	1180 (69.7%)	1144 (70.4%)	36 (53.7%)		163 (59.7%)	129 (62.0%)	34 (52.3%)		1017 (71.6%)	1015 (71.6%)	2 (100%)	
Second wave_High transmission	148 (8.7%)	132 (8.1%)	16 (23.9%)		40 (14.6%)	24 (11.5%)	16 (24.6%)		108 (7.6%)	108 (7.6%)	0 (0%)	
Decline of the second wave_Low transmission	122 (7.2%)	118 (7.3%)	4 (6.0%)		21 (7.7%)	17 (8.2%)	4 (6.2%)		101 (7.1%)	101 (7.1%)	0 (0%)	
**Clinical data**												
Days of symptoms until sample collection ^b^	5 [4–8]	5 [4–8]	6 [4–10]	0.08	6 [4–9]	6 [4–9]	6 [4–10]	0.802	5 [4–7]	5 [4–7]	2 [1–3]	0.041

^a^ *p* values were calculated using the chi-square (χ^2^) command for qualitative variables and the Mann–Whitney U Test for quantitative variables. The values are expressed as numbers (%: percentage of the total) or as ^b^ median [IQR: interquartile range], as appropriate. ^c^ Health worker, State security forces, Driver, Traders, and independents; ^d^ Kidney disease, lung disease, HIV, and cancer. NA: not applicable. Statistically significant results (*p* < 0.05) are in bold.

**Table 2:** Ct value of SARS-CoV-2 as a risk factor for death in patients with symptomatic COVID-19 and hospitalized with COVID-19. Amazonas, Peru, during the period of 2020–2021.

		Patients with Symptomatic COVID-19 (Outpatient and Hospitalized)			Patients Hospitalized for COVID-19		
Variable		aHR	Valor *p*	95% CI	HRa	Valor *p*	95% CI
Ct value ^a^	High Ct (Ct_s_ ≥ 28.4)	Reference category			Reference category		
	Low Ct (Ct_s_ < 28.4)	1.61	0.067	0.97–2.67	1.82	0.03	1.06–3.12
Age in years		1.09	<0.001	1.07–1.11	1.07	<0.001	1.05–1.09
Male sex		2.32	0.004	1.31–4.13	1.33	0.346	0.74–2.40
Occupational group with high level of exposure to SARS-CoV-2		1.71	0.098	0.91–3.22	2.28	0.015	0.74–2.40
Days of symptoms	1–3 days	Reference category			Reference category		
	4–7 days	1.69	0.151	0.83–3.45	1.95	0.101	0.88–4.33
	8–15 says	2.22	0.033	1.07–4.62	1.38	0.436	0.61–3.11
Comorbidities	Diabetes	1.81	0.171	0.77–4.24	1.49	0.379	0.61–3.64
	Cardiovascular disease	0.82	0.565	0.43–1.59	0.61	0.153	0.31–1.20
Epidemic dynamics	Second wave decline	Reference category			Reference category		
	First wave	0.61	0.474	0.15–2.39	0.36	0.152	0.09–1.45
	Decline of the first wave	1.67	0.45	0.44–6.35	1.18	0.81	0.30–4.62
	Beginning of the second wave	0.83	0.736	0.29–2.42	0.66	0.44	0.23–1.90
	Second wave	2.92	0.064	0.94–9.04	2.12	0.19	0.69–6.53

^a^ Ct value targeting the ORF1ab gene; aHR: Adjusted Hazard Ratio; 95% CI: 95% confidence interval. Statistically significant results (*p* < 0.05) are in bold.

## Data Availability

The original contributions presented in this study are included in the article. Further inquiries can be directed to the corresponding author(s).
